# Frequency of myasthenic crisis in relation to thymectomy in generalized myasthenia gravis: A 17-year experience

**DOI:** 10.1186/1471-2377-4-12

**Published:** 2004-09-11

**Authors:** Ali Soleimani, Alireza Moayyeri, Shahin Akhondzadeh, Mohsen Sadatsafavi, Hamidreza Tavakoli Shalmani, Akbar Soltanzadeh

**Affiliations:** 1 Division of neurology, Shariati hospital, Tehran University of Medical Sciences, Tehran, Iran; 2 Research Development Center, Shariati hospital, Tehran University of Medical Sciences, Tehran, Iran; 3 Psychiatric Research Center, Roozbeh Hospital, Tehran University of Medical Sciences, Tehran, Iran

## Abstract

**Background:**

Myasthenic crisis is the most serious life-threatening event in generalized myasthenia gravis (MG) patients. The objective of this study was to assess the long-term impact of thymectomy on rate and severity of these attacks in Iranian patients.

**Methods:**

We reviewed the clinical records from 272 myasthenic patients diagnosed and treated in our neurology clinic during 1985 to 2002. Fifty-three patients were excluded because of unconfirmed diagnosis, ocular form of MG, contraindication to surgery, concomitant diseases and loss to follow-up. The Osserman classification was used to assess the initial severity of the disease. Frequency and severity of the attacks were compared between two groups with appropriate statistical tests according to the nature of variables. Multivariate logistic regression analysis was used to assess the predictors of myasthenic crisis in the group of patients without thymoma.

**Results:**

110 patients were in thymectomy group and the other 109 patients were on medical therapy. These two groups had no significant differences with respect to age at onset, gender, Osserman score in baseline and follow up period. 62 patients (28.3% of all 219 patients) had reported 89 attacks of myasthenic crisis. 20 patients of 62 (32%) were in thymectomy group and 42 (68%) were in the other group. There was significant difference between the two groups in number of patients with crisis (P = 0.001; odds ratio = 2.8 with 95% CI of 1.5 to 5.2). In addition, these attacks were more severe in group of non-thymectomized patients as the duration of ICU admission was longer and they needed more ventilatory support during their attacks. Regression model showed thymectomy and lower age at onset as two predictors of decrement in myasthenic crisis rate in non-thymomatous MG patients.

**Conclusions:**

It is suggested that frequency and severity of myasthenic attacks as important endpoints in evaluation of MG patients. Thymectomy seems to have a preventive role on rate and severity of these attacks.

## Background

Myasthenia Gravis (MG) is the mostly known autoimmune disease in human, mediated by autoantibodies against the nicotinic receptor of acetylcholine in neuromuscular junctions [1]. Two cardinal features of this disease are muscle weakness and fatigability. Although periorbital area contains the only affected muscles in some patients, generalized weakness develops in approximately 85% of patients, affecting bulbar and limb muscles as well as the neck extensors and the diaphragm.

Myasthenic crisis refers to a rapid deterioration in neuromuscular function with respiratory compromise due to ventilatory muscle insufficiency or weakness of upper airway musculature or both [1]. These attacks might be triggered by multiple factors, including infections, physical or emotional stresses, aspiration, electrolyte disturbances, changes in medications and inadvertent administration of non-depolarizing muscle relaxants or other drugs. In this state, respiratory function should be monitored closely for evidence of respiratory failure and ventilatory support should be initiated in the setting of emerging respiratory failure [2,3].

In general, four methods of treatment are currently in use [4]: anticholinesterase agents, immunosuppressives, surgical thymectomy, and short-term immunotherapies, including plasma exchange and intravenous immune globulin (IVIG). Since the first operation for MG in 1911 [5], thymectomy has become an increasingly accepted procedure for treatment of MG, as it can help to achieve complete clinical remission rate between 18% and 50% [6-9] and clinical improvement in majority of patients [10]. The rationale for thymectomy is that about 75% of MG patients have thymic abnormalities; of these, 85% have hyperplasia and 15% have thymoma [11].

Although there is no convincing evidence regarding the use of thymectomy in non-thymomatous MG patients [12,13], most authorities recommend this procedure in all patients of ages between puberty and 60's [14,15]. However, this is not a general idea.

Thymectomy effects on occurrence and intensity of respiratory crisis have not been studied yet. Concerning value of this life-threatening event in MG patients, we studied the role of thymectomy on long-term frequency and severity of these attacks.

## Methods

We reviewed the clinical records from 272 myasthenic patients diagnosed and treated in the neurology clinic of Shariati Hospital, Tehran, during 1985 to 2002. All included patients had positive history and physical examination, confirmed with positive Tensilon test and electromyography (with repetitive nerve stimulation test). Searching for thymomas, all patients had a chest computed tomography (CT) scan.

The following patients were excluded from the study: 3 patients with doubtful test results and unconfirmed diagnosis; 25 patients with ocular form of MG (as these patients have no presentation of crisis); 2 patients with thymoma (and two episodes of crisis in the previous year), but contraindicated for surgery because of coronary artery disease; 6 patients with thyroid function abnormalities (because of their known effects on induction and reduction of myasthenic crises); 2 patients with concomitant rheumatoid arthritis (and consumption of high doses of corticosteroids and immunosuppressives); and 15 patients lost from follow-up with incomplete data. 219 MG patients enrolled in the study.

Myasthenic crisis was defined as an attack that compels physicians to hospitalize the patient and carefully supervise patient's respiratory capacity [3]. In most of these conditions, ventilatory support was needed. However, sometimes patient could be managed without intubation as a result of spontaneous improvement or use of plasmapheresis or IVIG.

All included patients were eligible for surgery. Patients with CT confirmed thymoma underwent elective thymectomy as soon as possible. In the first visit after diagnosis confirmation in non-thymomatous patients, the advantages and drawbacks of operation (including risks of anesthesia in MG patients) had been explained to them and deciding between the two choices of thymectomy or conservative therapy had been left to the agreement between physicians and patients. No particular protocol had been established for these patients and physicians had only a consultant role for patients with no force on them.

For clinical assessment of the initial severity of the disease, the classification of Osserman [16] had been applied to all patients in the first visit and inserted into patient's clinical profile. In following visits, patients' condition has been referred to this initial assessment. Data were collected on demographic factors, disease course at the first year of onset (or the year before surgery in thymectomized patients), time of surgery, pathology report, number of myasthenic crises (post-op in thymectomized patients), precipitating factors for attacks, ICU admission and the need for respiratory support, plasmapheresis or IVIG in each attack. 12 patients in thymectomy group had history of myasthenic crisis before surgery that were not considered.

The participants' baseline characteristics and follow-up data were analyzed by Student's *t *test and analysis of variances (ANOVA) as parametric tests, Mann-Whitney *U *test as non-parametric test, and Pearson's chi-square test for qualitative differences (SPSS software, version 10.0). *P *values less than 0.05 were taken to indicate statistical significance. Continuous variables are shown as the mean ± standard deviation (SD) and non-parametric variables with their median and range. Odds ratios and relative risks with 95% confidence intervals (95% CI) were calculated to assess the proportional risk of crisis between two groups.

In the subgroup of patients without thymoma, multivariate logistic regression analysis was used to assess the relationship between frequency of myasthenic crisis and several risk factors such as sex, start age of the disease, Osserman score and thymectomy. According to its percentile, start age was divided into three categories as bottom one-third (under percentile 33), middle one-third (between percentiles 34 and 66), and top one-third (over percentile 67).

## Results

110 patients underwent thymectomy (76 by trans-sternal and 34 by trans-cervical approaches) and 109 patients were on conservative therapy. Baseline characteristics and follow-up periods are shown in Table 1. Time interval between onset of myasthenic symptoms and thymectomy ranged from 1 to 192 months with median of 12. Mean age at thymectomy was 30.3 ± 12.8 years. During follow-up period, each patient had about 13 visits on average.

**Table 1:** Characteristics of thymectomized patients just before thymectomy and non-thymectomized patients at the first year of the diagnosis

	***Thymectomized patients (n = 110)***	***Non-thymectomized patients (n = 109)***	***P value***
Age at diagnosis (years)	29.2 ± 13.7	33.0 ± 15.9	0.059
Gender (male/female)	41/69	53/56	0.089

Osserman Score* = I	0	0	
Osserman Score = IIa	46	48	
Osserman Score = IIb	37	50	
Osserman Score = III	25	11	
Osserman Score = IV	2	0	
Follow up period (years)	6.4 ± 4.3	7.9 ± 5.6	0.134

* Mann-Whitney U test showed no significant difference between two groups (Z = |1.421| & P = 0.155)

Totally, 62 patients in both groups experienced 89 attacks of respiratory failure during their follow-up, which accounts for 28.3% of the study population. As it is shown in Table 2, 18.2% of thymectomized patients (20 of 110) and 38.5% of non-thymectomized patients (42 of 109) had history of these attacks. The difference between two groups was significant in this regard. Additionally, Odds ratio for being affected twice or more among patients on conservative therapy was 4.2 (with 95% CI of 1.1 to 16.7). Three patients in thymectomy group had history of crisis during post-op period in the hospital. Main triggering factors for myasthenic crises were lack of compliance to the drugs, pneumonia, and unknown causes Table 3.

**Table 2:** relation between myasthenic crisis frequency and thymectomy

	***Thymectomized patients (n = 110)***	***Non-thymectomized patients (n = 109)***	***P value***
Patients with myasthenic crisis (n = 62)	20	42*	0.001
Episodes of myasthenic crisis (n = 89)	25	64^†^	0.016

* This group had more probability of having crisis with an odds ratio of 2.8 (with 95% confidence interval of 1.5 to 5.2)

^†^Myasthenic crises were more prevalent in this group with a relative risk of 2.6 (with 95% confidence interval of 1.8 to 3.8)

**Table 3:** Characteristics of patients with myasthenic crisis in thymectomized and non-thymectomized patients*

	***Thymectomized patients (n = 20)***	***Non-thymectomized patients (n = 42)***
Age at diagnosis (years)	31.4 ± 10.6	30.5 ± 12.7
Age at first crisis (years)	33.5 ± 11.2	34.1 ± 11.3
Gender (male/female)	6/14	20/22
Triggering factors		
Pneumonia	3 (15%)	13 (30%)
Other infections	2 (10%)	4 (10%)
Aspiration	2 (10%)	3 (7%)
Stresses^†^	1 (5%)	2 (5%)
Drug intolerance	8 (40%)	10 (24%)
No obvious cause	4 (20%)	10 (24%)

* *There were no statistical differences between two groups*

† Refers to physical and emotional stresses as well as menstruation

To assess the severity of each attack, we considered duration of ICU admission (days), need to respiratory support and need to plasmapheresis or IVIG during crisis as indicating variables. As it is shown in Table 4 myasthenic crises were almost more severe in patients under conservative therapy. Two patients (both on conservative therapy) had passed away during respiratory failure (mortality rate = 2/89 = 2.2%) and these conditions were so protracted in some of patients that they were confined to ICU beds for more than one month.

**Table 4:** Severity parameters for each attack in patients with myasthenic crisis

	***Thymectomized patients (n = 25)***	***Non-thymectomized patients (n = 64)***	P value*
Median Days in ICU (Range)	7(2–38)	12(2–55)	0.044
Need to Ventilatory support	19 (76%)	59 (92%)	0.037
Need to Plasmapheresis or IVIG	7 (28%)	23 (36%)	0.476

* P values from chi-square test for assessment of difference between two groups

Pathology reports in thymectomized patients revealed 16 cases of thymoma. Fifty percent of these patients (8 of 16) had experienced crisis, which accounts for 40% (8 of 20) of thymectomized patients with history of crisis. Mann-Whitney *U *test revealed significant difference in this regard (P < 0.001). Severity indexes did not differ significantly between thymomatous and non-thymomatous patients Table 5.

**Table 5:** Characteristics of thymectomized patients with respect to pathology of thymus

***Thymus Pathology***	***N (% of 110)***	Age at diagnosis* (years)	***Sex (m/f)***	***Patients with Myasthenic crisis (%)***	***Number of Myasthenic crisis***	***Days in ICU (Median & Range)***	***Ventilatory support***
***Normal***	51 (46.4%)	29.7 ± 13.9	18/33	3 (5.9%)	3	3 & 2–4	3
***Hyperplasia***	43 (39.1%)	27.4 ± 15.2	14/29	9 (20.9%)	10	7 & 2–30	7
***Thymoma***	16 (14.5%)	37.3 ± 8.9	9/7	8 (50.0%)	12	9 & 3–38	9

* Analysis of variances indicated that age at diagnosis in thymomatous patients was significantly higher than others (P = 0.007). There was no statistically significant difference regarding other variables.

Totally, there were 203 patients without thymoma. Regression analysis in this subgroup of patients revealed that group of non-thymectomized patients as well as two third of patients with higher ages at the beginning of the disease were more prone to respiratory attacks Table 6.

**Table 6:** logistic regression model for predictors of myasthenic crisis among patients without thymoma (n = 203).

***Predictors***	***Odds Ratio***	***Lower 95% CI***	***Upper 95% CI***	***P value***
Start age (years)	1.016	0.996	1.038	0.085
Sex (male)	0.926	0.482	1.778	0.612
Thymectomy (performed)	0.234	0.113	0.484	<0.001
Osserman classification (score III)	1.269	0.410	3.928	0.529
Start age category (one third of younger patients)	0.272	0.097	0.759	0.021

## Discussion

Persuasive evidence for thymectomy in non-thymomatous generalized myasthenic patients is not on hand. In an attempt to establish standards in this regard, American Academy of Neurology advised thymectomy as only an *option *to increase the probability of remission or improvement in these patients [13]. In this review, thymectomy was associated with a median relative rate of medication free remission of 2.1 and a relative rate of improvement of 1.7. However, these improvements were significant in only a small number of the studies reviewed and the majority did not show a significant benefit with thymectomy. In addition, none of the studies was randomized or used blinded outcome assessments, and in most thymectomy was performed in younger patients.

Surprisingly, none of these studies has considered myasthenic crisis frequency as their study endpoint. For instance, in the study of Cohen *et al. *in 1981, 15 of 28 thymectomized patients experienced 21 crises during follow-up period [17]. In another study, 13 of 27 patients with crisis had previous thymectomy, six with thymoma [18]. This study was the first one that sought thymectomy impact on occurrence of crisis.

In our experience, persistence on thymectomy for all myasthenic patients would not be so wisely. Response to thymectomy is highly variable among these patients. Method of gaining agreement between physicians and patients would bring some benefits. As it is shown in Table 3, rate of intolerance to drugs was lower among the patients who preferred medical therapy. However, it is notable that there is a general tendency among both physicians and patients towards operation in younger ages and in patients with more severe course. In this study, thymectomized patients were about 4 years younger on average and had higher Osserman scores at the beginning (Table 1); but the difference was not statistically significant because of large study population and dispersion of baseline characteristics.

When myasthenic crisis occurrence is considered as the main endpoint of treatment in myasthenic patients, thymectomy has a great impact. Patients on conservative therapy had risk of about 3-fold of thymectomized patients to encounter respiratory attacks. However, the overall frequency of myasthenic crisis among our study population could be considered more than similar reports [2,4], perhaps reflecting some unidentified racial differences or lower compliance of Iranian patients to continuous medications.

Some previous studies have shown the benefit of thymectomy in the control of symptoms of MG. It has been revealed that delay in surgery could worsen the prognosis of MG [19] and the chance of benefiting from thymectomy increases when the history of MG is short and the stage of the disease is early [20]. Concerning differences of thymectomy effects in thymomatous and non-thymomatous patients, several studies have been conducted previously, showing some discrepancies in the results [21-23]. According to this study, thymomatous patients are more prone to respiratory attacks compared with all other MG patients. However, their attacks are not drastically different in severity of complications.

During myasthenic crisis, Weakness of the respiratory muscles may be out of proportion to that of other skeletal muscles. In rare cases, ventilatory failure is the only clinically apparent manifestation of the disease [24,25]. In addition, sometimes prediction of incoming attack is very difficult. In the present study, a 27-year-old woman experienced an attack after 7 months of complete remission on no drug.

Generally, infection is the most common trigger for myasthenic crisis [1]. Higher rate of infection in non-thymectomized patients in this study could be logically due to higher amounts of immunosuppressives in the medication regimen of these patients that we could not evaluate in this study. However, concerning frequency of myasthenic crises, this reduction of immunosuppressives in the regimen of thymectomized patients could be interpreted as an important benefit of thymectomy in MG patients.

The main limitation of this study was its retrospective design and the lack of randomization. Apparently, within a period of 17 years, many changes have occurred to diagnostic and therapeutic facilities as well as knowledge and attitude of physicians. These changes could have significant consequences on the outcome of myasthenic patients. Although various operative approaches seem not to differ drastically [26], various types of immunosuppressives and dosage of them have important role in the prognosis of patients, which we did not assess them in this study. We also could not gather information about acetylcholine receptor antibodies, which play an important role in the pathophysiology of MG. Further studies concerning the role of these antibodies in the occurrence and severity of myasthenic crises are required.

## Conclusions

In conclusion, we suggest evaluation of various aspects of myasthenic crisis as an important endpoint in long-term assessment of generalized MG patients. Moreover, we showed that thymectomy has a preventive role on rate and severity of myasthenic attacks in age and disease severity matched groups of patients. However, this effect needs further evaluation in upcoming prospective studies.

## Competing interests

None declared.

## Authors' contributions

In advance, suggestion of the design of the study was from our professor AkS. Data extraction and initial analysis were done by AlS and HT. SA participated in the design and implementation of the study. AM performed additional analyses and wrote the first draft of the paper. MS and SA both had helpful and valuable comments in revising the paper. All authors read and approved the final manuscript.

## Pre-publication history

The pre-publication history for this paper can be accessed here:


